# Report of the Charity Hospital, New Orleans

**Published:** 1843-02

**Authors:** 


### REPORT OF THE CHARITY HOSPITAL, NEW ORLEANS.

We are indebted to Dr. Barton for a copy of this report. The whole number of patients admitted into the hospital, from January 1830, to January 1843, a period of thirteen years, is fifty-nine thousand two hundred and one. Of these, forty-one thousand eight hundred and twenty are derived from foreign countries, chiefly from Ireland, Germany, England and France. Of the States, Pennsylvania has furnished the largest number, next comes New York, then Virginia, then Massachusetts, &c. Of the forty-one thousand eight hundred and twenty patients from abroad, Ireland alone has furnished *one half!* During the year ending January, 1843, there were four thousand four hundred and four persons admitted. Of these, three thousand five hundred and sixty had resided in the city less than three years, eight hundred and forty-one over three years; three thousand five hundred and sixteen were discharged, and seven hundred and four died. The most frequent diseases of that year were intermittent, yellow and remittent fever; rheumatism; dysentery; ulcer; syphilis; mania-a-potu; diarrhœa; contusions, &c.; their frequency being in the order named. We regret to learn that the financial affairs of the institution are so deranged. Its support is derived from private and public donations, and from Legislative grants; some of the latter have not been as productive as was anticipated. The expenditures have far exceeded the receipts, and the administrators of the establishment enter upon a new year with a load of debt amounting to $60091,32, which at the end of the year will be increased to nearly $80,000. To remedy this large and constantly increasing deficit, the report proposes that a tax be levied on the passengers arriving at the port of New Orleans, viz: on steerage passengers from foreign ports, $2,00 per head; on deck and steerage passengers from the United States, fifty cents; on cabin passengers from foreign ports, $3,00; on those from the United States, $1,00; leaving the inhabitants of the city, who have so many other calls on their charity, entirely exempt. A law similar to this is already in force, but owing to a defect in its provisions, or in its administration, the receipts have fallen far short of the amount anticipated. The perfect reasonableness of the proposed capitation tax is, we think, sufficiently apparent, as it will to a great extent make the sources of the vast expenditure of the institution, subservient to its support. The annual income under the new tax will, in the opinion of the Board, afford a permanent fund for the corporation; and it is to be hoped that the Legislature will comply with the suggestion, and not suffer so noble a charity to be destroyed. "*We cannot* close its doors," says the report; "the sick, destitute and distressed *must have relief;* we dare not, if we were even so disposed, shut the door of charity against them." The four thousand five hundred patients annually received into this hospital are of those "who cannot expect aid elsewhere." God forbid that such aid should be withheld. C.

---

We beg leave to call the attention of our readers to the card of Messrs. Erringer & Co., on the 4th page of the cover. They are ingenious and faithful workmen, and will, we are sure, be able to please the most fastidious. C.

